# *N*-(2-Bromo­phen­yl)-1,3-selenazolo[5,4-*b*]pyridin-2-amine

**DOI:** 10.1107/S1600536813023969

**Published:** 2013-09-12

**Authors:** Zhou Bo, Huang Du Shu, Liu Wei, Zhou Mei Yun

**Affiliations:** a College of Science, Honghe University, Mengzi 661100, People’s Republic of China; b Department of Chemistry, Jinan University, Guangzhou 510632, People’s Republic of China

## Abstract

The mol­ecular structure of the title mol­ecule, C_12_H_8_BrN_3_Se, is built up from fused selenazolo and pyridine rings, linked to a 2-bromo­aniline group. In the crystal, pairs of mol­ecules are linked by N—H⋯N hydrogen bonds into dimers, forming eight-membered ring motifs.

## Related literature

For the bioactivity of organoselenium compounds, see: Garud *et al.* (2007 ▶); Ling *et al.* (2010 ▶); Plamen *et al.* (2010 ▶). For crystallographic studies of selenazolo derivatives, see: Plamen *et al.* (2004 ▶).
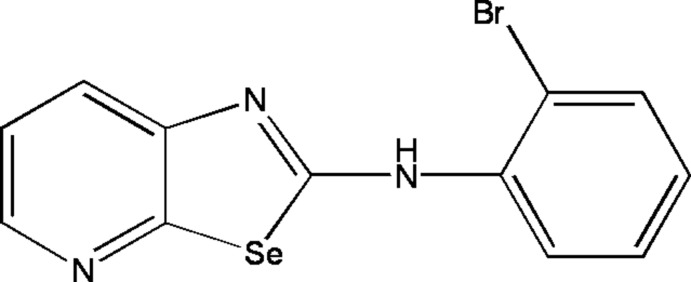


## Experimental

### 

#### Crystal data

C_12_H_8_BrN_3_Se
*M* *_r_* = 353.08
Monoclinic, 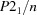

*a* = 12.5312 (5) Å
*b* = 7.4562 (3) Å
*c* = 13.8913 (5) Å
β = 112.331 (4)°
*V* = 1200.60 (8) Å^3^
*Z* = 4
Cu *K*α radiation
μ = 7.96 mm^−1^
*T* = 295 K
0.30 × 0.30 × 0.10 mm

#### Data collection

Agilent Xcalibur (Sapphire3, Gemini ultra) diffractometer
Absorption correction: multi-scan (*CrysAlis PRO*; Agilent, 2010) ▶ *T*_min_ = 0.199, *T*_max_ = 0.299
4420 measured reflections
1921 independent reflections
1779 reflections with *I* > 2σ(*I*)
*R*_int_ = 0.025

#### Refinement

*R*[*F* ^2^ > 2σ(*F* ^2^)] = 0.052
*wR*(*F* ^2^) = 0.132
*S* = 1.11
1921 reflections
155 parameters
H-atom parameters constrained
Δρ_max_ = 0.66 e Å^−3^
Δρ_min_ = −1.88 e Å^−3^

### 

Data collection: *CrysAlis PRO* (Agilent, 2010 ▶); cell refinement: *CrysAlis PRO*; data reduction: *CrysAlis PRO*; program(s) used to solve structure: *SHELXS97* (Sheldrick, 2008 ▶); program(s) used to refine structure: *SHELXL97* (Sheldrick, 2008 ▶); molecular graphics: *SHELXTL* (Sheldrick, 2008 ▶); software used to prepare material for publication: *publCIF* (Westrip, 2010 ▶).

## Supplementary Material

Crystal structure: contains datablock(s) I, New_Global_Publ_Block. DOI: 10.1107/S1600536813023969/fj2639sup1.cif

Structure factors: contains datablock(s) I. DOI: 10.1107/S1600536813023969/fj2639Isup2.hkl

Supplementary material file. DOI: 10.1107/S1600536813023969/fj2639Isup3.cml

Additional supplementary materials:  crystallographic information; 3D view; checkCIF report

## Figures and Tables

**Table 1:** Hydrogen-bond geometry (Å, °)

*D*—H⋯*A*	*D*—H	H⋯*A*	*D*⋯*A*	*D*—H⋯*A*
N3—H3⋯N2^i^	1.06	1.88	2.933 (4)	174

Symmetry code: (i) 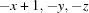.

**Fig. 1.**
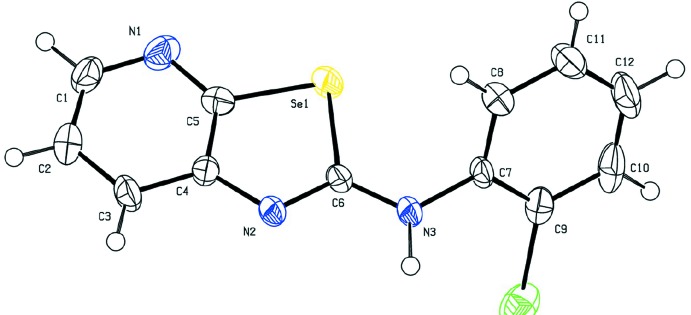
The molecular structure of the title compound in (I) showing the atom numbering Scheme. Displacement ellipsoids are drawn at the 50% probability level.

## supplementary crystallographic information

### 1. Comment

Since the discovery of the importance of Se as a microelement in bacteria and animals, and the function of the selenoenzyme glutathione peroxidase (GPx) as an antioxidant, the interest in organoselenium compounds has increased significantly (Garud, *et al.* 2007; Ling, *et al.* 2010; Plamen, *et al.* 2010,2004). The design and synthesis of organoselenium compounds, especially Se-containing heterocycles, are of our current interest. The title molecule (Fig.1) is built up from two fused rings, *viz*. selenazolo and pyridine, linked to 2-bromoaniline group. In the crystal, pairs of molecules are linked by N—H—N hydrogen bonds (H—N=2.933 Å) into dimers, forming eight- membered rings motifs.

### 2. Experimental

To a stirred solution of *N*-phenyllformamide (10 mmol) in toluene (100 ml) in an ice bath, Et_3_N (4.0 g, 40 mmol) and Se black powder were added. Then, phosgene (8 g of a 20% solution in toluene,) was added slowly over 30 min. An exothermic reaction took place. After complete addition, the suspension was heated under reflux for 10 h (TLC control). The mixture was filtered and washed with several portions of toluene, and then the filtrate was concentrated and afforded the raw isoselenocyanatobenzene. Isoselenocyanatobenzene was added to a stirred solution of 2-chloropyridin -3-amine (1.28 g, 10 mmol) in 2-propanol at room temperature, and the mixture was heated to reflux for 3 h. After filtration, the precipitate was collected as a yellow solid. The impure product was dissolved in CCl_2_H_2_ at room temperature. Colorless crystals suitable for X-ray analysis (90.4% yield) grew over a period of one week when the solution was exposed to the air.

### 3. Refinement

The structure was solved by direct methods and refined by least squares

method on F2 using the *SHELXTL* program package. All atoms were refined

anisotropically. Hydrogen atoms were placed at the calculated positions using

a riding model with C(aromatic)-H = 0.95 Å and *U*_iso_(H) = 1.2Ueq(C), and

with N—H = 0.95 Å and *U*_iso_(H) =1.5Ueq(N).

### Crystal data

**Table:**

C_12_H_8_BrN_3_Se	*F*(000) = 680
*M**_r_* = 353.08	*D*_x_ = 1.953 Mg m^−^^3^
Monoclinic, *P*2_1_/*n*	Cu *K*α radiation, λ = 1.54178 Å
*a* = 12.5312 (5) Å	Cell parameters from 1921 reflections
*b* = 7.4562 (3) Å	θ = 63.3–4.1°
*c* = 13.8913 (5) Å	µ = 7.96 mm^−^^1^
β = 112.331 (4)°	*T* = 295 K
*V* = 1200.60 (8) Å^3^	Prism, colorless
*Z* = 4	0.30 × 0.30 × 0.10 mm

### Data collection

**Table:**

Agilent Xcalibur (Sapphire3, Gemini ultra) diffractometer	1921 independent reflections
Radiation source: fine-focus sealed tube	1779 reflections with *I* > 2σ(*I*)
Graphite monochromator	*R*_int_ = 0.025
Detector resolution: 16.0288 pixels mm^-1^	θ_max_ = 62.8°, θ_min_ = 4.1°
ω scans	*h* = −14→14
Absorption correction: multi-scan (*CrysAlis PRO*; Agilent, 2010)	*k* = −8→8
*T*_min_ = 0.199, *T*_max_ = 0.299	*l* = −16→14
4420 measured reflections	

### Refinement

**Table:**

Refinement on *F*^2^	Primary atom site location: structure-invariant direct methods
Least-squares matrix: full	Secondary atom site location: difference Fourier map
*R*[*F*^2^ > 2σ(*F*^2^)] = 0.052	Hydrogen site location: inferred from neighbouring sites
*wR*(*F*^2^) = 0.132	H-atom parameters constrained
*S* = 1.11	*w* = 1/[σ^2^(*F*_o_^2^) + (0.1*P*)^2^] where *P* = (*F*_o_^2^ + 2*F*_c_^2^)/3
1921 reflections	(Δ/σ)_max_ < 0.001
155 parameters	Δρ_max_ = 0.66 e Å^−^^3^
0 restraints	Δρ_min_ = −1.88 e Å^−^^3^

### Special details

**Table:**

Experimental. CrysAlisPro, Agilent Technologies, Version 1.171.34.49 (release 20-01-2011 CrysAlis171 .NET) Empirical absorption correction using spherical harmonics, implemented in SCALE3 ABSPACK scaling algorithm.
Geometry. All e.s.d.'s (except the e.s.d. in the dihedral angle between two l.s. planes) are estimated using the full covariance matrix. The cell e.s.d.'s are taken into account individually in the estimation of e.s.d.'s in distances, angles and torsion angles; correlations between e.s.d.'s in cell parameters are only used when they are defined by crystal symmetry. An approximate (isotropic) treatment of cell e.s.d.'s is used for estimating e.s.d.'s involving l.s. planes.
Refinement. Refinement of *F*^2^ against ALL reflections. The weighted *R*-factor *wR* and goodness of fit *S* are based on *F*^2^, conventional *R*-factors *R* are based on *F*, with *F* set to zero for negative *F*^2^. The threshold expression of *F*^2^ > σ(*F*^2^) is used only for calculating *R*-factors(gt) *etc*. and is not relevant to the choice of reflections for refinement. *R*-factors based on *F*^2^ are statistically about twice as large as those based on *F*, and *R*- factors based on ALL data will be even larger.

### Fractional atomic coordinates and isotropic or equivalent isotropic displacement parameters (Å^2^)

**Table:**

	*x*	*y*	*z*	*U*_iso_*/*U*_eq_	
Se1	0.54412 (4)	0.09933 (7)	0.29605 (3)	0.0347 (2)	
Br1	0.25269 (5)	−0.30042 (7)	0.03920 (4)	0.0499 (2)	
C1	0.8930 (4)	0.1246 (6)	0.3969 (4)	0.0391 (10)	
H1	0.9579	0.1449	0.4594	0.047*	
N1	0.7879 (3)	0.1260 (5)	0.4012 (3)	0.0390 (9)	
C6	0.5142 (3)	0.0633 (5)	0.1513 (3)	0.0244 (8)	
N2	0.6067 (3)	0.0572 (5)	0.1289 (2)	0.0283 (7)	
C5	0.7000 (4)	0.1010 (5)	0.3106 (3)	0.0294 (9)	
C10	0.1263 (4)	−0.0412 (8)	0.1036 (3)	0.0467 (13)	
H10	0.0714	−0.1346	0.0929	0.056*	
N3	0.4086 (3)	0.0347 (5)	0.0812 (2)	0.0307 (8)	
H3	0.4002	0.0097	0.0038	0.11 (3)*	
C4	0.7091 (3)	0.0741 (5)	0.2149 (3)	0.0255 (8)	
C9	0.2266 (4)	−0.0716 (6)	0.0859 (3)	0.0320 (9)	
C3	0.8190 (4)	0.0674 (6)	0.2134 (3)	0.0354 (10)	
H3A	0.8303	0.0447	0.1507	0.042*	
C7	0.3078 (3)	0.0625 (6)	0.1015 (3)	0.0264 (8)	
C8	0.2863 (4)	0.2307 (6)	0.1358 (3)	0.0359 (10)	
H8	0.3407	0.3248	0.1466	0.043*	
C2	0.9120 (4)	0.0950 (6)	0.3064 (4)	0.0409 (11)	
H2	0.9885	0.0937	0.3079	0.049*	
C11	0.1872 (4)	0.2608 (8)	0.1538 (4)	0.0471 (12)	
H11	0.1743	0.3748	0.1779	0.056*	
C12	0.1068 (4)	0.1272 (9)	0.1372 (4)	0.0534 (15)	
H12	0.0379	0.1496	0.1486	0.064*	

### Atomic displacement parameters (Å^2^)

**Table:**

	*U*^11^	*U*^22^	*U*^33^	*U*^12^	*U*^13^	*U*^23^
Se1	0.0303 (3)	0.0541 (4)	0.0266 (3)	−0.00181 (18)	0.0184 (2)	−0.00480 (18)
Br1	0.0459 (4)	0.0368 (4)	0.0550 (4)	−0.0067 (2)	0.0055 (3)	0.0005 (2)
C1	0.028 (2)	0.044 (2)	0.034 (2)	0.000 (2)	−0.0008 (18)	−0.0034 (19)
N1	0.038 (2)	0.045 (2)	0.0263 (18)	−0.0011 (17)	0.0045 (16)	−0.0022 (16)
C6	0.0217 (19)	0.0271 (19)	0.0281 (19)	−0.0004 (15)	0.0135 (16)	−0.0024 (15)
N2	0.0222 (17)	0.0396 (18)	0.0269 (17)	−0.0023 (14)	0.0137 (14)	−0.0037 (14)
C5	0.034 (2)	0.028 (2)	0.028 (2)	0.0000 (16)	0.0148 (18)	−0.0021 (15)
C10	0.022 (2)	0.075 (4)	0.041 (3)	−0.009 (2)	0.010 (2)	0.016 (2)
N3	0.0199 (17)	0.047 (2)	0.0287 (17)	−0.0016 (15)	0.0134 (14)	−0.0077 (15)
C4	0.022 (2)	0.0283 (19)	0.0254 (19)	−0.0012 (15)	0.0082 (16)	−0.0003 (15)
C9	0.024 (2)	0.044 (2)	0.027 (2)	−0.0020 (18)	0.0082 (17)	0.0063 (17)
C3	0.024 (2)	0.048 (3)	0.039 (2)	0.0023 (18)	0.0179 (18)	0.0001 (19)
C7	0.0174 (18)	0.042 (2)	0.0230 (18)	−0.0001 (16)	0.0111 (15)	−0.0002 (16)
C8	0.031 (2)	0.042 (2)	0.039 (2)	0.0025 (19)	0.0177 (19)	−0.0041 (19)
C2	0.023 (2)	0.049 (3)	0.046 (3)	−0.0020 (18)	0.008 (2)	0.001 (2)
C11	0.038 (3)	0.067 (3)	0.040 (3)	0.015 (2)	0.018 (2)	−0.007 (2)
C12	0.028 (3)	0.098 (4)	0.041 (3)	0.010 (3)	0.021 (2)	0.005 (3)

### Geometric parameters (Å, º)

**Table:**

Se1—C5	1.886 (4)	N3—C7	1.410 (5)
Se1—C6	1.920 (4)	N3—H3	1.0555
Br1—C9	1.897 (5)	C4—C3	1.387 (6)
C1—N1	1.340 (7)	C9—C7	1.384 (6)
C1—C2	1.384 (7)	C3—C2	1.387 (6)
C1—H1	0.9500	C3—H3A	0.9500
N1—C5	1.334 (6)	C7—C8	1.404 (6)
C6—N2	1.309 (5)	C8—C11	1.375 (6)
C6—N3	1.328 (5)	C8—H8	0.9500
N2—C4	1.388 (5)	C2—H2	0.9500
C5—C4	1.390 (6)	C11—C12	1.372 (8)
C10—C9	1.388 (7)	C11—H11	0.9500
C10—C12	1.393 (8)	C12—H12	0.9500
C10—H10	0.9500		
			
C5—Se1—C6	83.98 (17)	C7—C9—C10	121.1 (4)
N1—C1—C2	123.6 (4)	C7—C9—Br1	119.4 (3)
N1—C1—H1	118.2	C10—C9—Br1	119.5 (4)
C2—C1—H1	118.2	C4—C3—C2	117.9 (4)
C5—N1—C1	115.4 (4)	C4—C3—H3A	121.0
N2—C6—N3	123.0 (3)	C2—C3—H3A	121.0
N2—C6—Se1	114.5 (3)	C9—C7—C8	118.3 (4)
N3—C6—Se1	122.3 (3)	C9—C7—N3	121.6 (4)
C6—N2—C4	113.9 (3)	C8—C7—N3	120.1 (4)
N1—C5—C4	125.7 (4)	C11—C8—C7	120.7 (5)
N1—C5—Se1	123.5 (3)	C11—C8—H8	119.6
C4—C5—Se1	110.7 (3)	C7—C8—H8	119.6
C9—C10—C12	119.5 (5)	C1—C2—C3	119.7 (4)
C9—C10—H10	120.3	C1—C2—H2	120.1
C12—C10—H10	120.3	C3—C2—H2	120.1
C6—N3—C7	123.2 (3)	C12—C11—C8	120.5 (5)
C6—N3—H3	117.4	C12—C11—H11	119.8
C7—N3—H3	118.6	C8—C11—H11	119.8
C3—C4—N2	125.6 (4)	C11—C12—C10	120.0 (4)
C3—C4—C5	117.5 (4)	C11—C12—H12	120.0
N2—C4—C5	116.8 (4)	C10—C12—H12	120.0
			
C2—C1—N1—C5	−1.6 (7)	C12—C10—C9—C7	0.3 (7)
C5—Se1—C6—N2	−0.2 (3)	C12—C10—C9—Br1	−179.6 (4)
C5—Se1—C6—N3	175.3 (4)	N2—C4—C3—C2	177.1 (4)
N3—C6—N2—C4	−174.2 (4)	C5—C4—C3—C2	−2.5 (6)
Se1—C6—N2—C4	1.3 (4)	C10—C9—C7—C8	0.0 (6)
C1—N1—C5—C4	0.1 (6)	Br1—C9—C7—C8	179.8 (3)
C1—N1—C5—Se1	−179.5 (3)	C10—C9—C7—N3	−178.3 (4)
C6—Se1—C5—N1	178.7 (4)	Br1—C9—C7—N3	1.5 (5)
C6—Se1—C5—C4	−0.9 (3)	C6—N3—C7—C9	−125.4 (4)
N2—C6—N3—C7	−172.0 (4)	C6—N3—C7—C8	56.4 (6)
Se1—C6—N3—C7	12.8 (6)	C9—C7—C8—C11	0.3 (6)
C6—N2—C4—C3	178.2 (4)	N3—C7—C8—C11	178.7 (4)
C6—N2—C4—C5	−2.1 (5)	N1—C1—C2—C3	0.9 (8)
N1—C5—C4—C3	2.0 (6)	C4—C3—C2—C1	1.2 (7)
Se1—C5—C4—C3	−178.4 (3)	C7—C8—C11—C12	−0.9 (7)
N1—C5—C4—N2	−177.7 (4)	C8—C11—C12—C10	1.1 (8)
Se1—C5—C4—N2	1.9 (4)	C9—C10—C12—C11	−0.8 (7)

### Hydrogen-bond geometry (Å, º)

**Table:**

*D*—H···*A*	*D*—H	H···*A*	*D*···*A*	*D*—H···*A*
N3—H3···N2^i^	1.06	1.88	2.933 (4)	174

Symmetry code: (i) −*x*+1, −*y*, −*z*.
